# *Campylobacter* culture fails to correctly detect *Campylobacter* in 30% of positive patient stool specimens compared to non-cultural methods

**DOI:** 10.1007/s10096-019-03499-x

**Published:** 2019-02-19

**Authors:** Janice E. Buss, Michelle Cresse, Susan Doyle, Blake W. Buchan, David W. Craft, Steve Young

**Affiliations:** 1 TECHLAB, Inc., Blacksburg, VA USA; 2 Wisconsin Diagnostic Laboratories, Medical College of Wisconsin, Milwaukee, WI USA; 3 Penn State Milton S. Hershey Medical Center and College of Medicine, Pathology and Laboratory Medicine, Hershey, PA USA; 4 TriCore Reference Laboratories, Albuquerque, NM USA

**Keywords:** *Campylobacter spp.*, Culture, Immunoassay, *Campylobacter upsaliensis*, Composite reference method

## Abstract

*Campylobacter* diagnosis is hampered because many laboratories continue to use traditional stool culture, which is slow and suffers false-negative results. This large multi-site study used a composite reference method consisting of a new FDA-cleared immunoassay and four molecular techniques to compare to culture. Prospectively collected patient fecal specimens (1552) were first preliminarily categorized as positive or negative by traditional culture. All specimens were also tested by EIA, and any EIA-positive or culture-discrepant results were further characterized by 16S rRNA qPCR, eight species-specific PCR assays, bidirectional sequencing, and an FDA-cleared multiplex PCR panel. The five non-culture methods showed complete agreement on all positive and discrepant specimens which were then assigned as true-positive or true-negative specimens. Among 47 true-positive specimens, culture incorrectly identified 13 (28%) as negative, and 1 true-negative specimen as positive, for a sensitivity of 72.3%. Unexpectedly, among the true-positive specimens, 4 (8%) were the pathogenic species *C. upsaliensis*. Culture had a 30% false result rate compared to immunoassay and molecular methods. More accurate results lead to better diagnosis and treatment of suspected campylobacteriosis.

## Introduction

Cases of *Campylobacter* spp.-associated gastroenteritis and diarrhea are increasing, not just in under-developed countries, but in developed countries such as the USA and Australia as well [1, 2]. There are now nearly one million cases of *Campylobacter* infection that are reported each year in the USA [3]. In developing countries, campylobacteriosis is endemic, with 8–45% of children being infected, whether they have diarrheal symptoms or not [4–6].

Since 2004, the annual incidence of *Campylobacter* infection in the USA detected only by culture had averaged 13.2/100,000 population. In 2015, campylobacteriosis was added to the nationally notifiable disease list and the “probable” case definition was revised to include cases detected by culture-independent diagnostic tests (CIDTs) [7]. After this change in detection criteria, the reported incidence rate rose to 17.4/100,000 population [2, 7]. This increase raised concerns about the accuracy of culture and previous prevalence estimates [8–12]. Other reports have found that culture’s sensitivity ranged from 60 to 76% [9, 13]. These results are worrisome, because they are occurring in the face of decreasing numbers of infections with other food-borne pathogens [1, 14] and an increasing number of antibiotic-resistant *Campylobacter* strains [15]. The trend towards resistance to fluoroquinolones has been noted by the World Health Organization and has led it to list *Campylobacter* spp. as among the top 12 global priority pathogens for which new treatments are needed [16, 17]. In 2013, the CDC reported resistance to ciprofloxacin in almost 25% of *Campylobacter* tested [18].

Accurate diagnosis of *Campylobacter* is important clinically. While most cases of campylobacteriosis are self-limiting and require no intervention other than oral rehydration therapy (ORT), severe cases can require antibiotics and carry the risk of major complications, such as Guillain-Barré syndrome paralysis [19, 20]. For patients with serious diarrheal symptoms, gastroenteritis caused by *Campylobacter* is not distinctive enough to guide clinical choices of treatment. As a result, empiric antibiotics (a fluoroquinolone such as ciprofloxacin, or azithromycin) are often prescribed while the patient and doctor await results of stool specimen culture [21]. Such antibiotic treatment is a considerable gamble as it can increase the patient’s risk for acquiring *Clostridioides difficile* infection, can be unnecessary if the cause is viral, or, if the suspected infection is actually shiga-toxin producing *E. coli* O157, can put the patient at risk for development of hemolytic uremic syndrome [22]. The improper use of unnecessary or inappropriate antibiotics also contributes to antibiotic resistance.

A serious problem for diagnosis of campylobacteriosis is continued reliance on culture methods to detect *Campylobacter* spp. in human stool. Growth of *Campylobacter* is slow, requiring 48–72 h, and involves specialized growth medium and chambers for microaerophilic growth [23]. Culture accuracy is limited by the tendency of *Campylobacter* to die erratically during handling, and by the difficulty of detecting microscopic colonies among competing fecal flora [10, 24]. Placing specimens in transport medium is thought to prolong organism survival, but length of successful storage is poorly defined [25]. The smallest amount of *Campylobacter* that culture can detect among competing fecal flora has not been reported. This information is necessary for the fundamental correlation of the numbers of bacteria detected by culture (and culture-independent tests) with clinical diarrheal symptoms. Such an estimate will be also be useful for study of protective immunity [26] or asymptomatic carriage [5, 6] of *Campylobacter* spp., especially in endemic settings.

Correct diagnosis of *Campylobacter* infection is important for antibiotic avoidance whenever possible. Despite its history of use, stool culture has long been suspected of failing to accurately identify a significant number of *Campylobacter* infections [23]. Such false-negative results can mean that the patient may continue to receive ineffective antibiotic treatment (e.g., if bacteria are fluoroquinolone resistant) [21]. False-positive results from conventional culture on plates containing antibiotics, when a candidate colony among fecal flora is not actually *C. jejuni* or *C. coli*, have also been reported and led to the development of multiple methods to optimize accurate recognition of colonies [27, 28]. Although uncommon, such false-positive results can encourage continued potentially unsafe antibiotic treatment and, more importantly, cut short the search for the causative pathogen.

This study provides a previously unavailable estimate of how many *C. jejuni* or *C. coli* can be detected in fecal cultures and tests how soon viability losses in Cary-Blair transport medium affect detection of *Campylobacter-*positive specimens by culture. In addition, diarrheal patient specimens were first tested by culture then confirmed as correct or re-assigned as true-positive or true-negative specimens by screening with five methods based on different principles (enzyme immunoassay and four molecular tests). Both the analytical and clinical studies established that the accuracy of the rapid immunoassay and molecular methods were equivalent and demonstrated the limitations of culture.

## Materials and methods

### Enumeration of *Campylobacter* in fecal specimens

Type strains of *C. jejuni* (ATCC 33560) and *C. coli* (ATCC 33559) were grown in pre-reduced BHI broth (BD Biosciences, San Jose, CA) containing 4% fetal bovine serum, 0.5% each trypticase and protease peptone, 0.0125% sodium pyruvate, and 0.0125% sodium bisulfite. Flasks were incubated at 37 °C in an anaerobic jar containing a CampyGen™ gas generating system sachet (Hardy Diagnostics, Santa Maria, CA). Growth of the bacteria was monitored by turbidity at OD_600_ and incubation stopped after 48 h or before OD_600_ values reached ~ 0.4. This OD_600_ typically equated to ≥ 10^7^ CFU/mL. Standard plate counts were performed on duplicate plates.

Six other *Campylobacter* species (Table 1) were grown according to vendor instructions to characterize reactivity of the immunoassay and species-specific qPCR. Table 1 shows the strain numbers used by different vendors.

**Table 1:** Identification numbers of equivalent strains of *Campylobacter* spp. and genes targeted for species-specific qPCR

Species	Strain Number			Gene target
	ATCC^a^	NCTC^b^	CCUG^c^	
*C. jejuni*	**33560** ^d^	11,351	11,284	*hipO*
*C. coli*	**33,559**	11,366	11,283	*cadF*
*C. upsaliensis*	43,954	11,541	**14,193**	*cpn60*
*C. lari*	35,221	11,352	**23,947**	*cpn60*
*C. helveticus*	51,209	12,470	**54,661**	*cpn60*
*C. fetus*	27,374	10,842	**6823A**	*cpn60*
*C. hyointestinalis*	35,217	11,608	**14,169**	*cpn60*
*C. concisus*	33,237	11,485	**13,144**	*cpn60*

^a^ATCC is American Type Culture Collection, Manassas, Virginia, USA

^b^NCTC is National Collection of Type Cultures, Salisbury, UK

^c^CCUG is Culture Collection of the University of Gothenburg, Gothenburg, Sweden

^d^Bold is indicated for strain numbers used in this study

At the same time as the plates for analytical counts (AnaeroGRO™ Campylobacter-selective Agar, Hardy Diagnostics) were prepared, a second set of broth dilutions was made by diluting 100 μL of turbid broth into 0.9 mL of a *Campylobacter-*negative fecal pool. The pool was made from diarrheal patient surveillance specimens that had been tested by the *Campylobacter* EIA and 16S rRNA PCR. A control plate with no *Campylobacter* added to the fecal pool was included in each experiment to help identify non-*Campylobacter* colonies. The streaked plates were examined visually at 48 h for colonies resembling those from pure *Campylobacter* cultures. Gram stain and microscopy was used to confirm that the selected colonies had *Campylobacter* morphology and were gram negative. If either of the duplicate plates at a particular dilution had 1 or more *Campylobacter* colony present, that dilution was considered fecal-culture positive. The analytical counts were then used to calculate the CFU/mL present in the second set of fecal dilutions.

### Cary-Blair sample stability study

One milliliter of *C. jejuni* broth culture prepared as above was mixed with 1 ml of the negative fecal pool. Duplicate 2-fold serial dilutions of the pool were prepared in additional fecal pool. Each dilution of the fecal curve was diluted 1:4 into Cary-Blair transport medium (Thermo-Fisher Scientific) per manufacturer’s instructions. The fecal dilutions in Cary-Blair medium were stored at 2–8 °C for 96 h with daily fecal culture in duplicate occurring at time zero and every 24 h. Simultaneous analytical colony counts of the broth were performed as above.

### Clinical studies

De-identified fecal specimens, submitted for stool culture and not collected specifically for this study, were collected between May 2017 and September 2017 at three independent clinical sites. All specimens were obtained from stool submitted for routine testing from patients who presented with clinical symptoms of gastroenteritis and diarrhea. All specimens from the TriCore Reference Laboratories and Wisconsin Diagnostic Laboratories were received in Cary Blair or C&S transport media. Specimens from Hershey Medical Center were received as fresh specimens (72 specimens) or in transport medium (82 specimens). Specimens were stored at 2–8 °C and within 24 h of receipt and were tested using that site’s standard laboratory *Campylobacter* culture method. All sites used Campylobacter-selective agar (Campylobacter CVA Agar with 10% sheep’s blood and antibiotics) for the fecal cultures and grew cultures in a 42 °C microaerophilic environment for 72 or 48 h (Hershey Medical Center). Fecal cultures with colonies showing *Campylobacter*-like colony morphology were tested by conventional Gram stain and biochemical activity assays. Positive colonies were sub-cultured to expand the isolate and identified with MALDI-TOF. In one instance, a colony that had been deemed *Campylobacter*-positive, expanded, and then tested by MALDI-TOF contained no detectable *Campylobacter* when assayed by EIA or any of the molecular methods. In Part One of the study, after removing an aliquot for culture, 876 specimens were quickly frozen and shipped to TECHLAB for immunoassay testing. At TECHLAB, the specimens were thawed only once, and samples were taken for EIA and DNA extraction. In Part Two of the study, 676 specimens were cultured and without delay tested with the immunoassay directly at the clinical sites. For both parts One and Two, only the specimens with positive or discrepant results compared to culture were tested at TECHLAB using the molecular methods of the Composite reference method described below. In both parts One and Two, the results of the immunoassay agreed completely with those of the molecular methods.

### Composite reference method

For immunoassay, an FDA-cleared, rapid, membrane-based EIA (the *CAMPYLOBACTER QUIK CHEK*™ test, TECHLAB, Inc., Blacksburg, VA) was performed according to package insert instructions. All molecular and EIA results were required to agree in order to confirm or re-assign a culture-tested specimen as true-positive or true-negative. It should be noted that this EIA assay agreed fully with the molecular tests described below.

A rigorous panel of molecular methods was assembled to determine if discrepant specimens truly contained *Campylobacter* or not. This composite reference method (CRM) consisted of qPCR for *Campylobacter* spp. 16S rRNA, 8 species-specific PCR assays, bidirectional sequencing, and the xTAG® GPP panel (Luminex Corporation, Austin, TX) as well as the immunoassay.

For the molecular assays, DNA was extracted and purified from all positive and discrepant fecal specimens using NucliSENS® easyMag (BioMérieux, Marcy-l’Étoile, France). The xTAG® GPP assay panel was run according to the manufacturer’s instructions. Specimens that contained *C. upsaliensis* were adjudicated by the three other molecular assays, as the xTAG® GPP test does not detect *C. upsaliensis*. PCR primers and assays for 16S rRNA of *Campylobacter* spp. and eight *Campylobacter* species-specific PCR assays using the genes noted in Table 1 were developed and validated with pure cultures and with bacteria spiked into negative fecal specimens [29, 30]. For bidirectional sequencing, DNA was amplified by 16S PCR [10] and amplified bands sent to the Biocomplexity Institute of Virginia Tech (Blacksburg, VA) for analysis. The species-specific PCR and sequencing results agreed fully.

## Results

### Culture-detectable levels of *C. jejuni* and *C. coli* in human stool

Amounts of *Campylobacter* in patient specimens have been previously estimated to be 10^6^–10^9^ CFU/mL [31, 32]; however, the limiting number of *Campylobacter* per milliliter that culture can detect in stool has not been reported. Estimating that threshold required that two simultaneous assessments be made. One test used visual detection of *Campylobacter* colonies from serial dilutions of spiked fecal cultures; the second quantified the CFU/mL in the pure bacterial culture used for spiking.

From seven independent bacterial slurries (5 *C. jejuni* and 2 *C. coli*), the detection thresholds for *Campylobacter* by culture spanned from 0.3–5 × 10^6^ CFU/mL. The *C. coli* results overlapped with the *C. jejuni* range; averages of detection limits for each set of slurries were 2 × 10^6^ and 1.2 × 10^6^ CFU/mL for *C. jejuni* and *C. coli*, respectively. These results suggested that culture should detect either *C. jejuni* or *C. coli* in patient specimens down to roughly 10^6^ CFU/mL. The detection threshold for the EIA is 8.4 × 10^4^ CFU/mL for *C. jejuni* and 7.7 × 10^5^ CFU/mL for *C. coli*.

An additional series of tests addressed how long microaerophilic *C. jejuni* retained viability (ability to be cultured) when refrigerated specimens were stored in Cary Blair transport medium. These data are central to the accuracy of culture for fecal specimens that must be shipped from a clinic or office to reference labs for testing. At the time of preparation of sample dilutions, the stock broth had an initial concentration of 4.8 × 10^7^ CFU/mL. When these (2-fold to 516-fold) dilutions were incubated, *C. jejuni* was detected on the plates streaked with the 32-fold dilution (equivalent to 1.5 × 10^6^ CFU/mL), similar to the results above. However, in an identical dilution series made after holding the stock for 24 h in Cary Blair medium at 2–8 °C, only the 2-fold dilution (equivalent to 2.4 × 10^7^ CFU/mL) grew visible colonies, a 16-fold (94%) loss of culturable organisms. This detection threshold was maintained out to 96 h, when testing was discontinued. This indicated that within 1 day, culture from stool in Cary Blair medium risked missing specimens with less than 10^7^ CFU/mL *C. jejuni.* These analytical studies were augmented by a large study with actual patient specimens.

The clinical accuracy of culture was assessed at three major laboratories by testing 1552 prospective stool specimens submitted for routine analysis. Age and gender information was available for all 1552 patients. Patient ages ranged from less than 1 to 100 years. Of the 1552 patients, 15.7% were ≤ 18 years, 38.7% were females, and 61.3% were males. No difference in culture performance was observed based on patient age or gender.

Among the 1552 specimens, culture yielded 35 positive results while the immunoassay and 4 molecular assays produced 47 positive results (Table 2). One of the 35 culture-positive specimens was EIA- and CRM*-*negative. The total number of discrepant results was 14. Testing with 4 molecular methods also affirmed that the 1 culture-positive but immunoassay*-*negative result was negative and that the 34 concordant culture- and EIA-positive specimens were positive. These true-positive specimens included 31 *C. jejuni-* and 3 *C. upsaliensis-*positive specimens. Of the 13 additional culture-negative but immunoassay*-*positive discordant specimens, the CRM confirmed that all 13 were true-positive, with 12 *C. jejuni*-positive and 1 *C. upsaliensis*-positive. These 13 culture-negative specimens were from patients with diarrhea and symptoms of gastroenteritis, showing that culture had missed clinically positive *Campylobacter* specimens.

**Table 2:** Clinical performance of culture

*N* = 1552	EIA+	EIA−	^a^CRM+	CRM−
Culture +	34	1	34	1
Culture−	13	1504	13	
Sensitivity	72.3%			
Specificity	99.9%			

^a^CRM refers to the EIA and four molecular assays of the composite reference methods

When compared to the immunoassay, culture correctly identified 34 positive specimens, but called 13 true-positive specimens as negative and one true-negative specimen as positive, giving a sensitivity of 72.3% and a specificity of 99.9%. Compared to the four molecular reference methods, the EIA had 100% correlation on all discrepant specimens (Table 2). Overall, culture produced false-negative (13) or false-positive (1) results in 29% of 48 specimens. Culture results with specimens collected at the three clinical laboratories were similar and gave an ~ 30% rate of incorrect assignment (Table 3). Rates of incorrect culture results for the 676 never-frozen specimens that were immunoassay-tested at the three laboratory sites and for the 876 specimens that were frozen and tested at TECHLAB were equivalent.

**Table 3:** Culture results at three clinical sites

	Cultures	Culture positive	^a^CRM results	% Culture incorrect
Site no. 1	367	5	8 positive	3/8 = 37.5%
Site no. 2	219	6	8 positive 1 negative	2/8 = 25% [3/9 = 33.3%]^b^
Site no. 3	966	24	32 positive	8/32 = 25%
Average				29.1% [31.9%]

^a^CRM refers to the EIA and four molecular assays of the composite reference methods

^b^Values in brackets include the one culture false positive

The failure of culture to correctly identify specimens was not because of low numbers of *Campylobacter* in the specimens. Six of the 13 false-negative culture specimens had *Campylobacter* 16S rRNA (with Ct negative cutoff of > 40) values that were below a Ct of 30 (approximately 3 × 10^6^ CFU/mL). Two specimens had Ct values below 26. These ostensibly culture-negative specimens contained large numbers of *Campylobacter*. This extensive range of bacterial burden in false-positive results of culture is similar to results reported previously [9, 33].

Of note, species-specific PCR and bidirectional sequencing indicated that four specimens from symptomatic patients contained pathogenic *C. upsaliensis*, representing 8.5% of the 47 true-positive specimens. The 8 species-specific PCR assays did not detect any other *Campylobacter* spp. in this cohort of patients. Overall, the prevalence rate of *Campylobacter* infection during the months of May to September 2017, as determined by the CRM tests, was 3.0% (47/1552), whereas prevalence based on culture only was 2.3% (35/1552).

## Discussion

This large prospective study agrees with previous studies on the poor sensitivity of *Campylobacter* culture [9, 34, 35]. Over one-quarter of the specimens that were confirmed to contain *Campylobacter* DNA and bacterial antigen had been classified as culture-negative. Moreover, the rapid loss of *Campylobacter* viability in clinical specimens stored in transport medium as found in this study suggests that low levels of live *Campylobacter* in actual patient specimens may not survive to be detected by culture. Based on the PCR Ct values of some specimens, even reasonably high numbers of bacteria may not be recovered by culture.

A strength of the clinical evaluation phase of this study was the exclusive use of freshly collected, all-comers patient specimens without resort to banked frozen samples. This approach utilized *Campylobacter*-positive specimens that reflect natural prevalence rates during the summer season in three geographically separate regions of the USA. Further, the molecular and immunological characterization of the specimens required simultaneous agreement of all CRM methods to accurately arbitrate results that were discrepant with culture. This rigorous multi-pronged approach was necessary because faulty culture results could make a single comparator assay appear inaccurate. The complete agreement of these combined reference methods clearly showed that culture incorrectly identified a significant number of specimens and produced a sensitivity of only 72%.

Typical laboratory culture methods are optimized for *C. jejuni* and *C. coli* and are not set up to detect additional pathogenic *Campylobacter* species like *C. lari* and *C. upsaliensis* [10, 36]. In this study, the immunoassay and molecular methods confirmed that *C. upsaliensis* was present in ~ 10% of all clinically positive specimens. *C. upsaliensis* is known to be able to cause human disease [37], but its clinical importance has been recognized by only a few studies [36].

These studies are subject to several limitations. Although our cultures could detect *C. jejuni* and *C. coli* in fecal specimens that contained an average of about 1 million bacteria per gram of stool, the *Campylobacter* concentrations detected with fecal cultures spanned a 15-fold range. Although *Campylobacter*-specific agar utilized by many clinical laboratories was used for the study, other specialized solid media with alternative antibiotics might make colony detection among competing fecal flora more precise. In addition, a pooled mixture of multiple *Campylobacter-*negative but diarrheal stools was used as the sample matrix for culture. Diarrheal stool is more realistic than healthy stool, but the effect of the pooled specimens’ true sources of gastroenteritis on *Campylobacter* detection is unknown. In actual clinical situations, variations among patient fecal specimens or use of antibiotics will likely make the culture thresholds from individuals differ as well. Another limitation is that only the 48 discrepant and positive specimens were tested by the 4 molecular reference methods. However, all 1552 specimens were tested by the EIA, an assay that showed 100% agreement with the molecular methods on the discrepant and positive specimens. Requiring that all 5 CRM methods had to agree for a specimen’s results to be resolved was used to strengthen the validity of the results.

The findings of this report provide practical information on culture-independent methods that will be useful for both small and large diagnostic laboratories as well as provide unexpected results on under-reported pathogenic species that are important for physicians and epidemiologists.

These results underline the limitations of culture as the gold standard for *Campylobacter* detection [9, 38, 39] and suggest that culture-independent tests should have a role in diagnostic testing. This is important clinically because continued reliance on culture may hold back the adoption of new, more accurate assays. For antibiotic resistance testing, epidemiological studies, or required state or national reporting, culture will still be required. In these situations, the rapid time-to-result detection by an immunoassay or newer molecular tests will permit the > 97% of the specimens that are negative to be screened and separated within a time frame when the true-positive specimens should still contain viable *Campylobacter* spp. and can be reflexed to culture for further testing. Improving the detection rate for species that are often overlooked may show that the true prevalence of *Campylobacter* spp. infection is higher than currently recognized by culture alone [40], especially for *C. upsaliensis*.
